# Differential gene expression provides leads to environmentally regulated soybean seed protein content

**DOI:** 10.3389/fpls.2023.1260393

**Published:** 2023-09-18

**Authors:** Julia C. Hooker, Myron Smith, Gerardo Zapata, Martin Charette, Doris Luckert, Ramona M. Mohr, Ketema A. Daba, Thomas D. Warkentin, Mehri Hadinezhad, Brent Barlow, Anfu Hou, François Lefebvre, Ashkan Golshani, Elroy R. Cober, Bahram Samanfar

**Affiliations:** ^1^ Ottawa Research and Development Centre, Agriculture and Agri-Food Canada, Ottawa, ON, Canada; ^2^ Department of Biology, Ottawa Institute of Systems Biology, Carleton University, Ottawa, ON, Canada; ^3^ Canadian Centre for Computational Genomics, Montréal, QC, Canada; ^4^ Brandon Research Centre, Agriculture and Agri-Food Canada, Brandon, MB, Canada; ^5^ Crop Development Centre, University of Saskatchewan, Saskatoon, SK, Canada; ^6^ Morden Research and Development Centre, Agriculture and Agri-Food Canada, Morden, MB, Canada

**Keywords:** RNA-seq, differential expression, soybean, asparagine, seed protein, amino acid metabolism

## Abstract

Soybean is an important global source of plant-based protein. A persistent trend has been observed over the past two decades that soybeans grown in western Canada have lower seed protein content than soybeans grown in eastern Canada. In this study, 10 soybean genotypes ranging in average seed protein content were grown in an eastern location (control) and three western locations (experimental) in Canada. Seed protein and oil contents were measured for all lines in each location. RNA-sequencing and differential gene expression analysis were used to identify differentially expressed genes that may account for relatively low protein content in western-grown soybeans. Differentially expressed genes were enriched for ontologies and pathways that included amino acid biosynthesis, circadian rhythm, starch metabolism, and lipid biosynthesis. Gene ontology, pathway mapping, and quantitative trait locus (QTL) mapping collectively provide a close inspection of mechanisms influencing nitrogen assimilation and amino acid biosynthesis between soybeans grown in the East and West. It was found that western-grown soybeans had persistent upregulation of asparaginase (an asparagine hydrolase) and persistent downregulation of asparagine synthetase across 30 individual differential expression datasets. This specific difference in asparagine metabolism between growing environments is almost certainly related to the observed differences in seed protein content because of the positive correlation between seed protein content at maturity and free asparagine in the developing seed. These results provided pointed information on seed protein-related genes influenced by environment. This information is valuable for breeding programs and genetic engineering of geographically optimized soybeans.

## 1 Introduction

Soybean (*Glycine max* [L.] Merr.) is one of the most important legume crops worldwide for use as human food and livestock feed. Soybean seeds contain the highest protein of any legume, which makes protein content a key quality attribute (Natarajan et al., 2013; Huang et al., 2019). In symbiosis with rhizobia, soybean fixes atmospheric nitrogen into more biologically available forms of nitrogen. Nitrogen fixation gives soybeans a valuable role in sustainable agricultural practices by reducing the need for nitrogen fertilizers and reducing incomplete nitrogen conversion/capture, which pollutes the surrounding environment (air, soil, and water). As the global population rises, strategic agricultural planning requires optimization of crops for different environmental conditions in order to produce adequate yields with acceptable levels of high-quality seed protein.

For more than two decades, observations have been made that soybeans grown in western Canada have lower (~1%–5%) seed protein content than eastern-grown soybeans. In 2022, the average eastern soybean protein content was 40.3%, while the western-grown soybeans had an average protein content of 38.9% (Canadian Grain Commission, 2022). In general, western Canadian soybean-growing regions have lower seasonal precipitation, sandier soils, shorter growing seasons with a longer photoperiod, and cooler temperatures, all of which contribute to reduced seed quality and are attributed to difficulties in successfully growing soybeans. Seed protein and oil contents are complex quantitatively inherited traits that are influenced by the combination of genotype and environment (Wang et al., 2019). Soybeans from western Canada have been observed to spend more time in the vegetative stages of development compared to eastern-grown counterparts and less time dedicated to flowering and seed development (Ort et al., 2022). Seed protein content is a key factor in soybean seed quality measures; profits are significantly impacted for farmers who grow soybeans in suboptimal environmental conditions. Further, as climates change and populations increase, it is of great economic and agricultural importance to make better use of the western and northern growing regions of Canada.

Soybean agronomic productivity is measured in part by seed composition, specifically regarding the total seed content of two major seed storage biomolecules: protein and oil. Generally, protein and oil contents have an inverse relationship in soybean seed; as oil increases, protein decreases, and vice versa (Breene et al., 1988; Clemente and Cahoon, 2009). Expression of genes involved in seed protein and oil is most highly expressed during the middle and late stages of development (Severin et al., 2010). Genes involved in fatty acid synthesis and elongation (*fad2*, *lox*, and *kcs*) contribute to the differences seen between high-protein-low-oil and low-protein-high-oil cultivars, while seed protein content differences are influenced by transcription factors (including *abi3* and *lec2*), and sugar transporter *SWEET10a* plays a role in both protein and oil accumulation (Wang et al., 2020; Peng et al., 2021). In a recent study, lipid and carbohydrate metabolism were found to be differentially expressed high-protein and high 11S soybeans in comparison to their low-protein and low-11S counterparts grown in the same conditions (Hooker et al., 2023). High protein requires the plant to direct a significant amount of nitrogen assimilates to the seeds. Total amino acid content is positively correlated with seed protein content in soybeans (Zhang et al., 2018). It has been observed that protein content at the time of soybean seed maturity is positively correlated with free asparagine in developing seeds (Hernández-Sebastià et al., 2005; Pandurangan et al., 2012). This relationship has also been observed in other crops, such as barley and maize (Dembinski and Bany, 1991; Lohaus et al., 1998). Soybean seed protein accumulation is controlled in part by the biosynthesis of nitrogenous assimilates in source leaves. Biologically available inorganic nitrogen in the soil (ammonium NH_4_^+^, nitrite NO_2_^−^, and nitrate NO_3_^−^) must be reduced to ammonia (NH_3_) before assimilation into amino acids (Lea and Miflin, 1980; Lea et al., 1990). However, there is a gap in understanding the mechanisms underlying the accumulation of nitrogen assimilates in the developing seed; it is unclear whether nitrogen assimilate supply is directed by the mother plant or if the developing seed has an intrinsic capacity for storage protein synthesis (Hernández-Sebastià et al., 2005). In large-seeded species like beans (*Phaseolus limensis* L.), seed size is sufficient enough that they have a vascular bundle, which allows for the direct distribution of nutrients from the mother plant to the developing seed (Vinogradova and Falaleev, 2012); however, further exploration into these processes in soybeans is needed.

RNA-sequencing (RNA-seq) and differential expression (DE) are powerful tools for functional genomics and transcriptomics. Comparing gene expression between two genetically identical samples in two different environmental conditions allows for a snapshot of the active and inactive genes directly influenced by the environment. Downstream analyses of the resulting DE genes give valuable information on the pathways and systems that are influenced by a given environment. Gene ontology (GO) and pathway mapping databases are regularly updated with novel and/or more robust information that can process large lists of genes to provide multi-perspective functional analysis. Quantitative trait locus (QTL) analysis uses variable quantitative traits and genotypic information to make correlations between the two. QTL mapping is useful for identifying molecular loci influential of a given biological pathway and offers information about locus location and linkage. Collectively, there are over 550 protein and oil QTLs known and available in SoyBase (www.soybase.org) distributed over all 20 chromosomes, but there are higher proportions falling on chromosomes 5, 15, and 20 (Wang et al., 2019). By uncovering DE genes with key functional roles in seed protein and/or oil development, avenues for genetic engineering of soybeans become more effective for breeding agriculturally sustainable soybean crops. It is hypothesized that soybeans grown in western growing regions are differentially expressing some of their seed protein-related genes when compared to those grown in the East. The objective of this study was to investigate the differential gene expression between soybeans grown in East and West Canada to uncover the key metabolic pathways potentially influencing seed protein content. To do this, RNA-seq and DE data were collected in 2019 spanning 10 soybean varieties, three experimental locations (West), and one control location (East).

## 2 Methods

### 2.1 Soybean lines

Ten soybean genotypes were selected as a representation of the range of seed protein content observed in Canada. Soybean lines are listed from the lowest to highest seed protein content, with line 1 having the lowest average protein content and line 10 having the highest average protein content. The soybean lines used in this study were all developed at the Ottawa Research and Development Centre by Agriculture and Agri-Food Canada: X5583-1-041-5-5 (line 1), AC Harmony (Voldeng et al., 1996a) (line 2), AAC Halli (line 3), 90A01 (Cober et al., 2006) (line 4), Maple Amber (line 5), OT13-08 (line 6), OT14-03 (line 7), AAC Springfield (line 8), Jari (line 9), and AC Proteus (Voldeng et al., 1996b) (line 10).

### 2.2 Planting and growth

Planting was performed in 2019 in replicated trials across four locations: Ottawa Ontario (latitude 45.39°, longitude −75.72°), Morden Manitoba (49.18°, −98.08°), Brandon Manitoba (49.86°, −99.98°), and Saskatoon Saskatchewan (52.15°, −106.57°). Seeds were planted in quadruplicate at the mid-end of May in a 4 × 5 rectangular lattice arrangement at a density of 50 seeds per m^2^, and appropriate crop management practices were taken at each site. For additional information on planting, see Cober et al. (2023).

### 2.3 Tissue collection and seed composition assessment

Young trifoliate leaf tissue was collected in triplicate from soybeans at the R5 (Pedersen and Licht, 2014) stage of maturity from otherwise healthy-looking plants. Tissue was flash-frozen in liquid nitrogen in the field immediately upon harvest, and samples were stored at −80°C. Western samples were shipped overnight on dry ice and immediately stored at −80°C until RNA extraction. From each plot, measurements for total seed protein and oil contents were performed using an Infratec 1241 Grain Analyzer (FOSS North America, Eden Prairie, MN, USA) at the Agriculture and Agri-Food Canada Ottawa Research and Development Centre. For additional phenotypic information, see [25].

### 2.4 RNA extraction

RNA extractions using SPLIT Total mRNA Extraction Kit (Lexogen, Vienna, Austria) were performed on approximately 200 mg of crushed leaf tissue from each sample according to the manufacturer’s instructions. RNA quality was tested using a NanoDrop™ 2000 Spectrophotometer (Thermo Fisher Scientific, Waltham, MA, USA), agarose gel electrophoresis (1%), TapeStation 4200 RNA ScreenTape (Agilent, Santa Clara, CA, USA), and 2100 Bioanalyzer (Agilent, Santa Clara, CA, USA) at Génome Québec (Montréal, QC, Canada) and the Ottawa Research and Development Centre (Ottawa, ON, Canada). RNA integrity number (RIN) values of at least 6.5 and a Q30 score of at least 36 were selected for library preparation. Spike-in RNA variants (SIRVs) (Lexogen, Vienna, Austria) were integrated into the RNA samples as controls to monitor and compare key parameters (such as sensitivity and quantification); the E0 SIRV mix was used, containing 69 different isoform variants with known sequences at equal molar concentrations.

### 2.5 RNA-seq library preparation, alignment, read mapping, read counting, and DE

Paired-end sequencing was carried out using the Illumina HiSeq 4000 platform (Illumina, San Diego, CA, USA) at Génome Québec to create cDNA libraries for each sample. RNA-seq data were assessed using dupRadar (Sayols et al., 2016) (v3.16, Biberach an der Riß, Germany; Bioconductor, R) for duplication rate quality control. Normalization of reads was carried out at the individual sample level using edgeR (Robinson et al., 2010) (v3.16, Parkville, Victoria, Australia). Exploratory data analysis of normalized reads was performed using R.

QualiMap (García-Alcalde et al., 2012) (v2.2.1, Berlin, Germany) was used as a quality control step for sequence data feature alignment (genes and transcripts). Preseq (Daley et al., 2020) (v3.1.1, Los Angeles, CA, USA; Bioconductor, R) was used to estimate the number of distinct reads from each RNA-seq library. RSeQC (Wang et al., 2012) (v4.0.0, Nanjing, China; Bioconductor, R) was the primary tool used for comprehensive evaluation of the RNA-seq read data through the calculation of semantic read distribution of a sample, distance between reads, duplication presence, and junction saturation.

The Canadian Centre for Computational Genomics uses an in-house framework program, GenPipes (Bourgey et al., 2019), to perform the following major processing steps. Trimmomatic (Bolger et al., 2014) (v0.36, Jülich, Germany) was used to remove adaptor sequences and low quality score bases (phred score <30). Trimmed reads were aligned to the soybean genome (Glycine_max_v2.1, INSDC Assembly GCA_000004515.4, Jul 2018), using STAR (Dobin et al., 2013) (v2.7.7a, Menlo Park, CA, USA) under the command –runMode alignReads after generating index files from the aforementioned genome. HTSeq (Anders et al., 2015) (v0.12.3, Heidelberg, Germany) was used to obtain read counts using the following options: “-m intersection-nonempty”.

### 2.6 Differential gene expression analysis and candidate gene identification

DE analysis was performed using DESeq2 (Love et al., 2014) (v3.16, Heidelberg, Germany) with negative Binomial GLM fitting and Wald statistics: nbinomWaldTest. To transform expression data to be expressed as log_2_FC, “ashr” (Stephens, 2017) was used. All datasets were trimmed to an adjusted p-value <0.01. For DE analysis, Ottawa samples were used as the control, and the three western locations were each used as the experimental data; the log_2_ fold change (log_2_FC) difference in expression data reflects a change occurring in the western-grown relative to eastern-grown cultivars. Identical genotypes were compared for each DE analysis, and comparisons were not made across different genotypes.

All DE datasets were amended with the corresponding information from the SoyBase Genome Annotation Source v2.0 (https://soybase.org/genomeannotation/), which includes annotation data from BLASTP, TAIR10, GO, Panther, PFAM, and KOG for all genes. In this study, both top-down and bottom-up analyses were used to assess DE genes between East and West. “Top-down” and “bottom-up” are descriptive terms for the direction of data analysis. To clarify, the top-down analysis uses the holistic dataset and investigates the DE genes from a bird’s-eye perspective without any specific functional selection—i.e., which genes (and their ontologies) are DE between East and West at the given cut-off criteria (p-value <0.01, |log2FC| ≥ 1.5). The top-down approach was used to holistically search the DE data for the most consistent DE genes between East and West; DE was cross-compared across all 30 datasets for most of the consistently (30/30 datasets) DE genes. The bottom-up analysis describes a different approach to the data: an investigation in which we search only for genes involved in one specific pathway of interest (the Asp-Ala-Glu pathway). The bottom-up approach was used to search through the DE data to identify genes with “asparagine”, “aspartate”, “alanine”, “glutamate”, and “oxaloacetate” as a component of their annotation (BLASTP, TAIR10, GO, Panther, PFAM, or KOG). With the use of a short bash script, all 30 DE datasets were searched for any gene with these keyword identifiers and were short-listed for pathway-specific analysis. The purpose of this bottom-up analysis was to provide a comprehensive investigation of DE genes within this pathway and provide insight into the underlying molecular mechanisms influencing low seed protein phenotypes in western Canada. A p-value <0.01 and a log_2_FC change in expression of at least 1.5 (|log2FC| ≥ 1.5) were considered significantly DE for both the top-down and bottom-up approaches. Genes that are DE in 15 (50%) or more datasets were selected for downstream analysis. Because the SoyBase annotation database uses BLAST descriptions from 2014, an up-to-date BLAST search was carried out on all resultant top-down and bottom-up genes and is included alongside the SoyBase annotation output in **Supplementary Table 1** and **Supplementary Table 2**.

### 2.7 GO analysis

For the top-down analyses, GO term enrichment was assessed using the SoyBase GO Term Enrichment Tool (https://soybase.org/goslimgraphic_v2/dashboard.php) for the genes commonly upregulated and commonly downregulated across all 30 DE datasets. Enrichment was calculated from the ratio of expressed DE genes for a particular GO term to the expected number of DE genes for said term based on the total number of known associated genes in the full GO database.

### 2.8 Heatmapping

Heatmapping was performed using Heatmapper (Babicki et al., 2016) using log-normalized read counts across all samples in this study calculated using R. Clustering was calculated using average linkage, and distance matrices were calculated using Pearson’s coefficient. The row Z-score normalizes expression data to improve visualization of heatmap data trends; this score is calculated by (gene expression value in sample of interest) − (mean expression across all samples)/(standard deviation) (Anders and Huber, 2010).

### 2.9 KEGG analysis

Gene IDs were converted to their corresponding National Center for Biotechnology Information (NCBI) ID numbers, which were then mapped using the Kyoto Encyclopedia of Genes and Genomes (KEGG) release v106.0 (https://www.kegg.jp/) for pathway enrichment using the Mapper Search functions, with *Glycine max* (gmx) as the organism identifier.

### 2.10 QTL analysis

Chromosome positioning data for all 20 *G. max* chromosomes were extracted from SoyBase GWAS-based QTL database for all seed protein and oil QTLs (https://soybase.org/GWAS/list.php). The positional information for each of the 59 bottom-up genes of interest was also extracted from SoyBase and used to determine which genes fall within large QTL regions or in very close proximity to single-point QTLs. With the use of MapChart v2.32 (Voorrips, 2002), QTL and gene positional information were mapped to each chromosome and color-coded to be most informative.

## 3 Results

### 3.1 RNA-seq analyses

**Figure 1:**
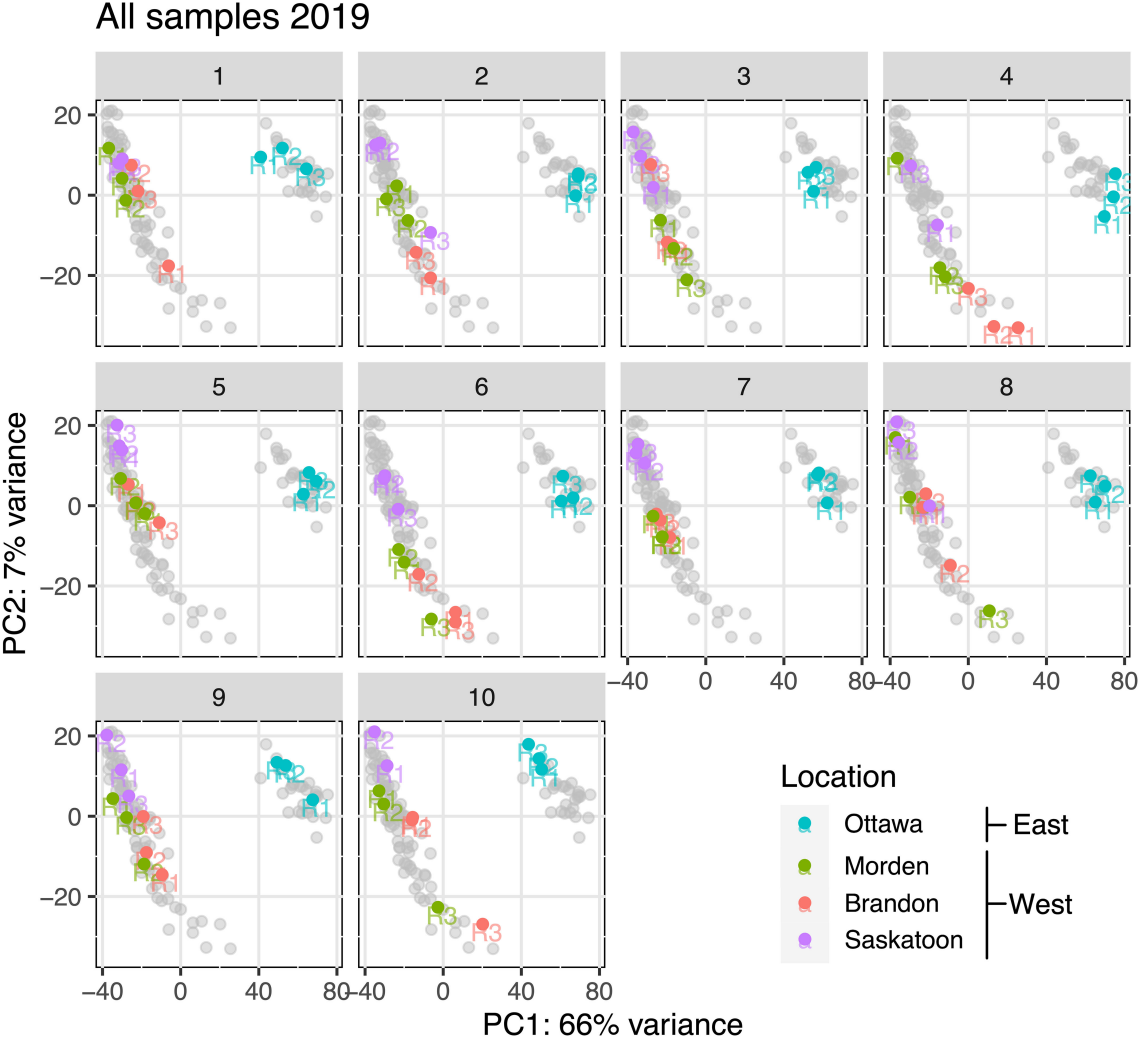
PCA for each soybean genotype (number at top of each panel) at each location based on total RNA-seq variance after removal of outliers and normalization. Soybean genotype number is represented above each corresponding PCA plot. PC1 is on the x-axis, and PC2 is on the y-axis. Gray points represent all the data points in other lines. Replicates within each line (R1, R2, and R3) are labeled at their corresponding points. PCA, principal component analysis (Hooker et al., 2022).

There was a total of 4,047,045,039 reads over all 87 RNA-seq datasets (10 lines, four locations, and three replicates per line). Missing replicates are Saskatoon line 1 replicate 1, Saskatoon line 4 replicate 2, and Saskatoon line 10 replicate 3, which did not pass quality control (QC) upon repeated attempts. **Table 1** summarizes the average seed protein and oil contents as a percentage of the total seed content. Included in **Table 1** is the cumulative read depth across the three replicates per sample. **Figure 1** shows the principal component analysis (PCA) of the transcriptome data for each replicate in East and West locations, organized by line (1–10). Across all samples, PC1 described a 66% variance, and PC2 described a 7% variance (**Figure 1**). From **Figure 1**, there is a clear distinction between East (cyan) and West (green, purple, and red) RNA-seq variability, indicating the RNA-seq data from the East are highly different than the data from the West, and the data from the three West locations cluster closely together, suggesting similar variability.

**Table 1:** Average seed protein and oil contents (given in percentage of total seed content at 13% moisture) and RNA-seq read depth for the three replicates for each soybean genotype in East and West locations in Canada in 2019.

Line	East			West								
	Ottawa			Morden			Brandon			Saskatoon		
	Protein	Oil	Read depth	Protein	Oil	Read depth	Protein	Oil	Read depth	Protein	Oil	Read depth
***1***	38.9	23.0	116,820,021	37.3	22.2	92,391,979	36.3	21.2	95,080,016	37.7	19.3	69,995,998
***2***	37.5	23.4	92,142,359	35.6	22.8	84,842,378	35.2	21.3	119,248,964	37.5	19.3	104,853,205
***3***	38.9	22.1	93,160,198	38.2	21.7	100,375,142	39.0	19.7	89,082,785	38.9	18.9	118,090,282
***4***	40.9	21.5	92,490,279	39.5	21.3	99,640,813	40.2	20.1	97,743,936	39.8	18.5	92,095,914
***5***	40.9	22.1	119,385,027	40.1	21.6	103,073,578	39.3	20.3	92,696,520	40.1	18.7	89,401,807
***6***	42.3	21.9	123,431,881	41.6	21.7	93,502,218	41.1	20.8	90,866,478	40.7	20.0	136,867,397
***7***	41.7	20.4	104,008,337	42.1	20.0	112,032,906	41.9	18.0	106,855,434	40.8	18.1	136,026,735
***8***	45.1	18.5	97,175,295	43.2	19.5	98,480,553	42.9	17.9	101,918,836	45.2	16.6	93,969,604
***9***	44.3	19.0	101,408,312	42.3	19.7	96,174,311	41.3	18.3	114,640,094	43.5	17.3	103,655,546
***10***	47.5	16.7	101,581,019	46.9	16.7	115,579,425	46.2	15.9	103,436,842	47.0	16.3	52,822,615

### 3.2 Top-down approach to DE analysis

#### 3.2.1 Upregulated genes

**Figure 2:**
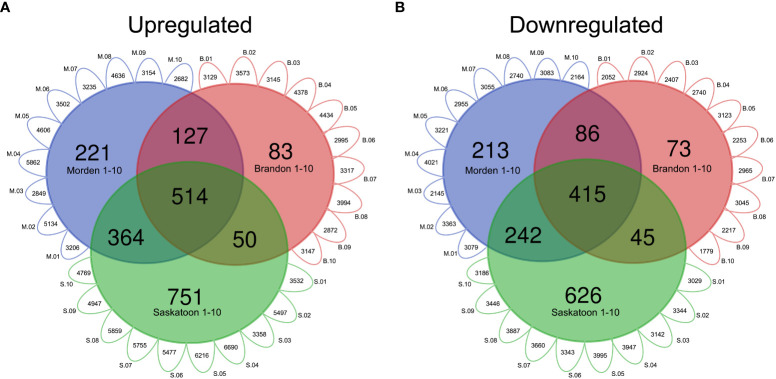
Venn diagrams of DE soybean genes in western locations relative to east. Modified Venn diagram of the number of genes in top-down DE analyses for each western location. Small exterior petals represent the number of **(A)** upregulated or **(B)** downregulated genes in each line-location pairwise comparison (p-value <0.01, log_2_FC 1.5). “M” (blue) represents Morden, “B” (red) represents Brandon, and “S” (green) represents Saskatoon. Gene IDs commonly up- or downregulated across all lines 1–10 per location were used to construct the Venn diagram. Common DE genes were found using the VLOOKUP function in MS Excel, and the Venn diagram was created using https://bioinformatics.psb.ugent.be/webtools/Venn/. DE, differential expression.

For the top-down analysis, genes identified to be significantly DE (|log_2_FC| ≥ 1.5, p-value <0.01) across all 30 datasets with the same orientation (up- or downregulated in the West compared to the East) were considered. For all DE genes for each line in each location, including unique IDs and commonly DE genes, see **Supplementary Table 1**. The top-down analysis from Brandon had a total of 34,984 instances of upregulated genes across all 10 genotypes, composed of 8,652 unique gene IDs, of which 774 were commonly upregulated across all 10 genotypes (datasets) (**Supplementary Table 1**). The Morden top-down analysis found a total of 38,866 instances of upregulated genes across all 10 lines, composed of a total of 8,620 unique gene IDs, 1,226 of which are commonly upregulated across all 10 lines in Morden (**Supplementary Table 1**). The Saskatoon top-down analysis had a total of 52,100 upregulated genes across the 10 datasets, composed of a total of 10,812 unique IDs, of which 1,679 were commonly upregulated across all 10 lines in Saskatoon (**Supplementary Table 1**). In total, 514 genes were commonly upregulated across all 30 East *vs.* West DE datasets (10 lines, three West locations) (**Supplementary Table 1**). **Figure 2A** shows a Venn diagram of the genes commonly upregulated across all lines; **Figure 2B** shows the commonly downregulated genes. The values given in the exterior “petals” of the Venn diagram represent the number of genes that were found to be either upregulated (**Figure 2A**) or downregulated (**Figure 2B**) in the individual East *vs.* West DE analyses, which were used to find common DE genes; this was performed to circumvent the infeasibility of presenting a 30-way Venn diagram with all possible combinations of line-location overlapping genes.

#### 3.2.2 Downregulated genes

In total, there were 29,826 instances of downregulation across all 10 lines in Morden, made up of 6,475 unique gene IDs and 956 genes commonly downregulated across the 10 lines (**Supplementary Table 1**). In Brandon, there were 25,505 instances of downregulation across all 10 lines, composed of 6,469 unique gene IDs, of which 619 were commonly downregulated across all 10 lines (**Supplementary Table 1**). There were 34,978 instances of downregulation in Saskatoon across all 10 DE datasets, composed of 6,931 unique gene IDs, and 1,328 of those genes were commonly downregulated across all 10 lines. There were 415 genes commonly downregulated across all 30 East *vs.* West datasets (**Figure 2B**; **Supplementary Table 1**).

#### 3.2.3 Gene ontology

**Figure 3:**
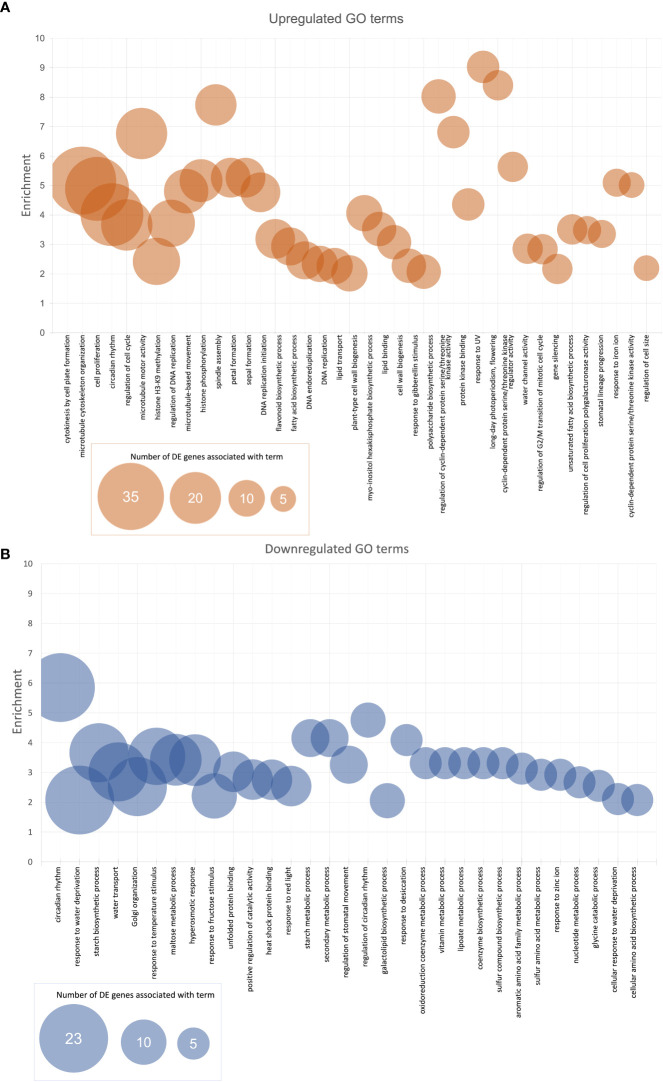
Enriched **(A)** upregulated and **(B)** downregulated BP and MF GO terms for top-down analysis of DE soybean genes between East and West. Enrichment was calculated by taking the proportion of the number of DE genes associated with a term and the expected number of genes associated with a term. Represented in the graphs are the GO terms with enrichment values of at least 2 (overrepresented by 100% or twofold) with a minimum of five expressed genes with GO terms in list. BP, biological process; MF, molecular function; GO, gene ontology; DE, differential expression.

After GO enrichment using the SoyBase GO Term Enrichment Tool, there were 730 GO terms (biological process (BP) and molecular function (MF)) associated with the genes consistently downregulated in the West and 815 GO terms associated with the genes consistently upregulated in the West (**Supplementary Table 1**). **Figure 3** shows the most highly enriched GO terms (BP and MF) across the genes consistently DE in the West across all lines. Bubble size indicates the number of DE genes in our list that are associated with a particular term. Enrichment was calculated using the proportion of the number of DE genes observed to be associated with a term divided by the number of genes expected to be among a list of the query size. GO terms graphed in **Figure 3** were selected based on an enrichment score over 2 (i.e., enriched by 100% or twofold) and at least five genes in our DE data with a given term included in their annotations. Terms are listed in order from the highest number of DE genes per term to the lowest (five genes, minimum). **Supplementary Table 1** summarizes the GO enrichment data for the persistently up- and downregulated genes. Included among the most enriched genes upregulated in the West are cytokinesis by cell plate formation (GO:0000911), spindle assembly (GO:0051225), microtubule motor activity (GO:0003777), response to UV (GO:0009411), long day photoperiodism (flowering) (GO:0048574), and cyclin-dependent protein serine/threonine kinase regulator activity (GO:0016538) (**Figure 3A**). Other noteworthy GOs from the upregulated genes include lipid-related ontologies, fatty acid biosynthetic process (GO:0006633), lipid transport (GO:0006869), and lipid binding (GO:0008289). Circadian rhythm (GO:0007623) was among the most enriched genes consistently up- and downregulated in the West (**Figures 3A, B**). Regulation of circadian rhythm (GO:0042752), secondary metabolic process (GO:0019748), starch metabolic process (GO:0005982), and starch biosynthetic process (GO:0019252) were among the topmost enriched GO terms from the list of genes downregulated in the West. Cellular amino acid biosynthetic process (GO:2000282), asparagine biosynthesis, aromatic amino acid family metabolic process (GO:0009072), and maltose metabolic process (GO:0000023) were noteworthy terms among the highly enriched downregulated genes (**Figure 3B**).

#### 3.2.4 KEGG pathway enrichment

**Figure 4:**
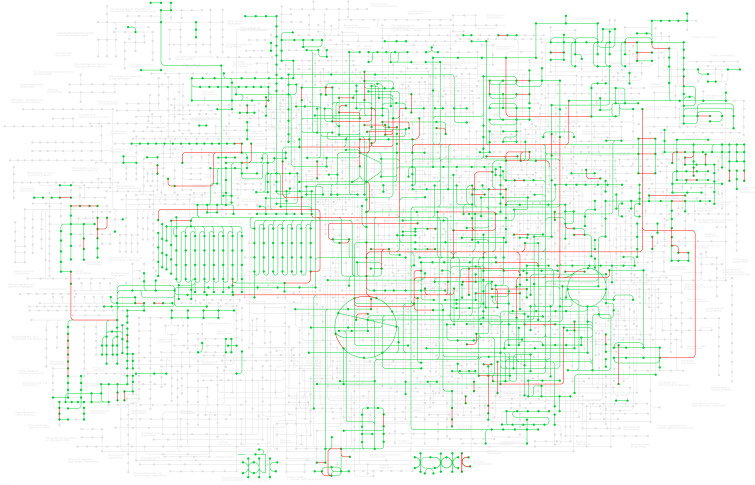
KEGG pathway enrichment of the up- and downregulated genes across all 30 DE datasets (|log_2_FC| ≥ 1.5, p-value <0.01*) across all known soybean metabolic pathways (gmx01100)*. Green highlight indicates known pathways in soybeans, and red highlight indicates pathways DE between eastern- and western-grown soybeans. For a larger image of this map, see **Supplementary Figure 1**. KEGG, Kyoto Encyclopedia of Genes and Genomes; DE, differential expression.

Gene IDs from up- and downregulated top-down DE analyses were converted to their NCBI gene ID number and then mapped to the *G. max* database (gmx) within the KEGG for pathway enrichment. Of the topmost enriched pathways, 109 genes mapped to the broad *G. max* metabolic pathway (gmx01100), 68 genes mapped to the biosynthesis of secondary metabolites pathway (gmx01110), 22 genes mapped to motor proteins (gmx04814), 13 genes mapped to plant hormone signal transduction (gmx04075), 12 genes mapped to circadian rhythm – plant (gmx04712), 11 genes mapped to carbon metabolism (gmx01200), and 10 genes mapped to the biosynthesis of cofactors (gmx01240). Other pathways of note include aromatic amino acid (phenylalanine, tyrosine, and tryptophan) biosynthesis (gmx00400; three genes), fatty acid metabolism (gmx01212; three genes), and sulfur-containing amino acid (cysteine and methionine) metabolism (gmx00270; three genes). The full list of enriched pathways and the genes that map to each are in **Supplementary Table 3**. **Figure 4** shows the enriched pathways within the broad *G. max* metabolic pathway (gmx01100); the red highlight indicates pathways DE between East and West grown soybeans.

### 3.3 Bottom-up approach to DE analysis

The top-down analysis showed significant enrichment of biosynthesis of secondary metabolites (gmx01110), which led to the downstream investigation of select sub-pathways. Among these sub-pathways, one pathway of interest was the alanine, aspartate, and glutamate (Ala-Asp-Glu) metabolism pathway (gmx00250), which was of particular interest because of the known relationship between nitrate assimilation during development and seed protein content at maturity (Hernández-Sebastià et al., 2005; Pandurangan et al., 2012). Asparagine synthesis and hydrolysis are components of the alanine, aspartate, and glutamate (Ala-Asp-Glu) metabolism pathway.

**Figure 5:**
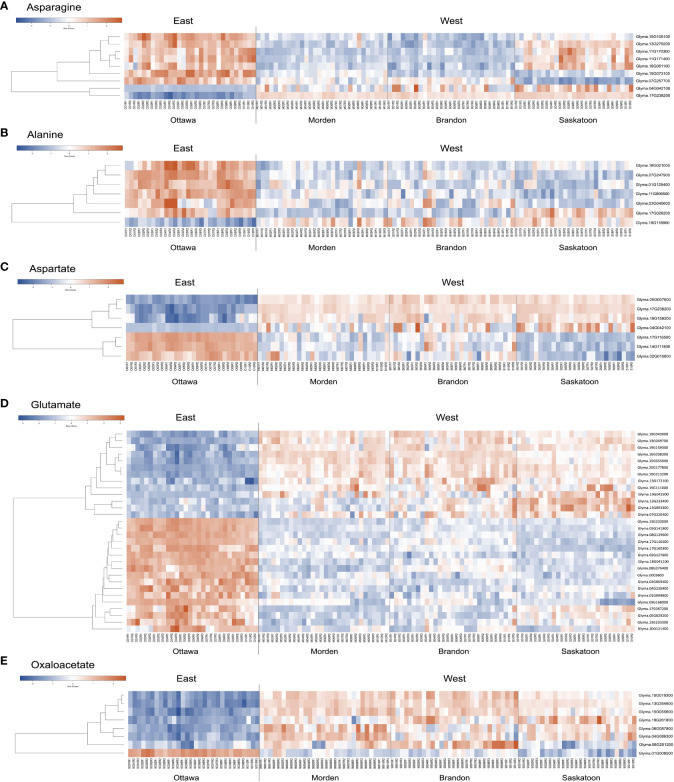
Heatmap of bottom-up soybean genes with ontologies related to **(A)** asparagine, **(B)** alanine, **(C)** aspartate, **(D)** glutamate, and **(E)** oxaloacetate. Heatmaps were created using Heatmapper (Babicki et al., 2016). On the left side of each heatmap are the relationships between the genes. On the right of each heatmap is the corresponding gene name. Replicate sample names are given at the bottom of the map. Clustering was calculated using average linkage, and distance measurements were calculated using Pearson’s coefficient. The row z-score for each heatmap is given; blue represents lower expression, and red represents higher expression. KEGG, Kyoto Encyclopedia of Genes and Genomes; DE, differential expression.

For the bottom-up analyses, significance criteria were loosened to include genes DE in a minimum of 15 of the 30 DE datasets, rather than all 30 datasets as used in the top-down analysis. This was to expand the DE data to include genes outside of the top-down lists. Significance criteria were maintained at a log_2_FC of at least 1.5 and p-value <0.01. DE data were searched for any DE genes with annotations including the terms “alanine”, “aspartate”, “glutamate”, “asparagine”, and “oxaloacetate”, which fit these significance criteria. **Supplementary Table 2** summarizes the log_2_FC in expression for all significantly DE genes with these annotations. **Figure 5** shows the relative expression data across all samples in this study as a heatmap; Pearson’s coefficient relationship between genes in each list was used to organize the heatmap. These heatmaps provide a visual summary of the expression data and the relationships between the genes, while the information in **Supplementary Table 2** provides the specific log_2_FC DE data (|log_2_FC| > 1.5).

#### 3.3.1 Asparagine-related genes

A total of nine unique asparagine-related gene IDs were identified (based on criteria of p-value <0.01 and a log_2_ fold change of 1.5 in a minimum 15 of 30 datasets) to be DE between East and West across all 30 datasets with 189 total instances of DE (**Supplementary Table 2**). Seven genes (*Glyma.07G257700*, *Glyma.13G279200*, *Glyma.15G073100*, *Glyma.15G105100*, *Glyma.11G171400*, *Glyma.11G170300*, and *Glyma.18G061100*) with asparagine synthetase (AS) annotations were found to be downregulated across all lines in all three western locations (**Figure 5A**; **Supplementary Table 2**). *Glyma.11G171400*, *Glyma.11G170300*, and *Glyma.18G061100* were all identified as AS (E.C.6.3.5.4) in *G. max*; these three genes were downregulated in Brandon and Morden, but DE in Saskatoon was limited to three instances, two of which were upregulated. Additionally, *Glyma.13G279200* and *Glyma.15G105100* had more instances of DE in Brandon and Morden than in Saskatoon. *Glyma.13G279200* encodes a stem-specific protein TSJT1, and *Glyma.15G105100* encodes a Wali7 domain-containing protein in *G. max*; both genes have PANTHER annotations of AS and TAIR10 identified the top *Arabidopsis* homolog is an aluminum-induced protein with YGL and LRDR motifs (AILP1). *Glyma.07G257700* was mostly found to be downregulated across the Saskatoon DE datasets (10), but also found to be downregulated in Brandon (2) and Morden (3); this gene had an NCBI identity of stem-specific protein TSJT1 in *G. max* based on model evidence. *Glyma.15G073100* was downregulated across all 30 DE datasets; this gene also encodes a stem-specific protein TSJT1 in *G. max* (**Supplementary Table 2**).

Two genes (*Glyma.17G238200* and *Glyma.04G042100*) identified as asparaginase (ASPG) (E.C.3.5.1.1) in *G. max* were found to be largely upregulated across all 30 datasets, with one (*Glyma.17G238200*, l-asparaginase) persistently upregulated across all 30 datasets and the other (*Glyma.04G042100*, asparaginase 2) upregulated across 19 datasets (**Figure 5A**; **Supplementary Table 2**).

**Figure 6:**
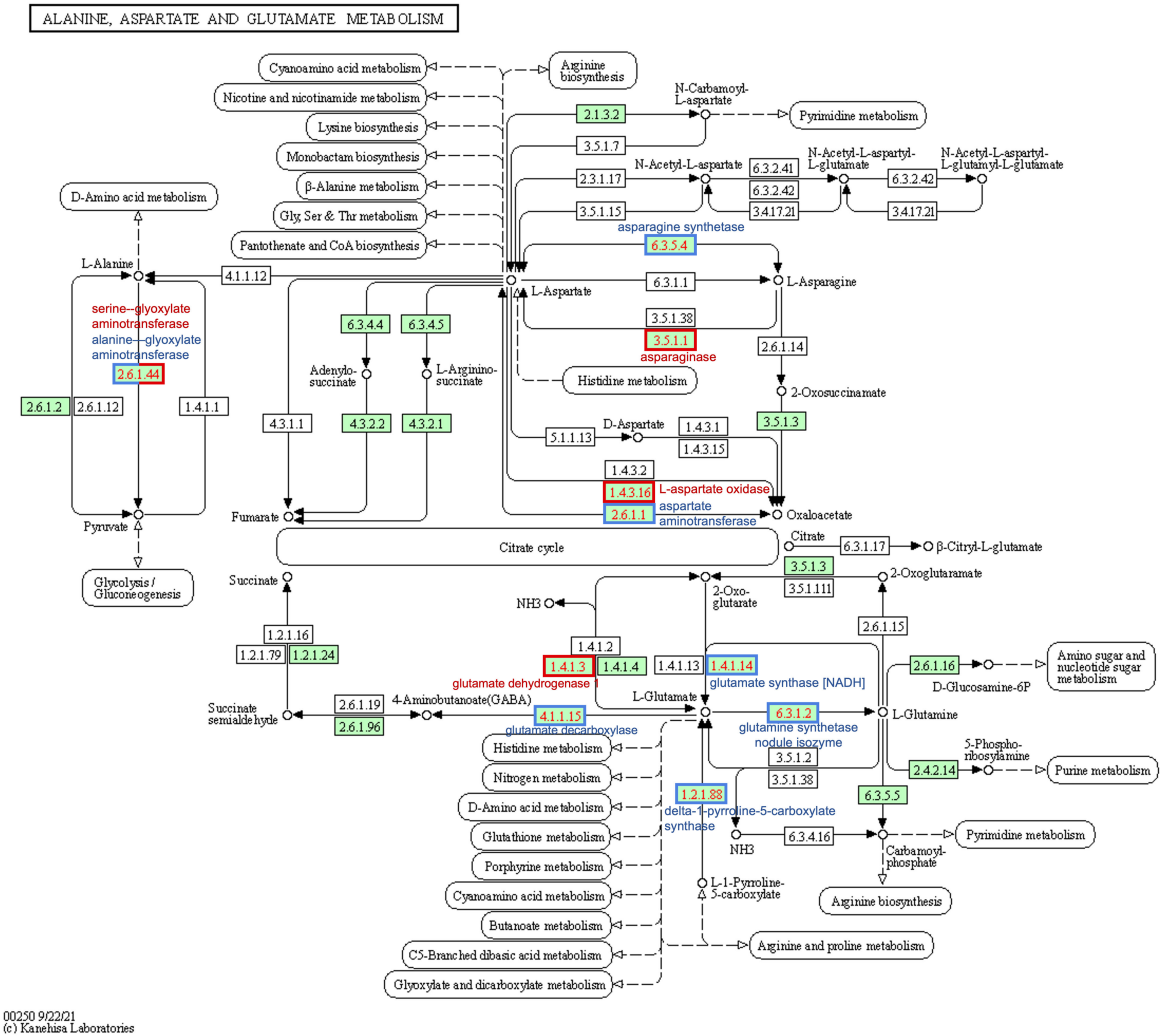
Differential expression of soybean genes represented on the KEGG alanine, aspartate, and glutamate metabolism pathway map (gmx00250). Numbers in boxes are Enzyme Commission numbers comprising one or more proteins. Green-colored boxes represent enzyme sets identified in soybeans (*Glycine max*); white boxes are not known in soybeans. Red-bordered boxes represent enzyme sets encoded by genes that are upregulated in western locations relative to east, and blue-bordered boxes represent downregulated genes. The half-blue-half-red box (2.6.1.44) was found to be upregulated for serine—glyoxylate and downregulated for alanine—glyoxylate aminotransferase; they fall under the same E.C. KEGG, Kyoto Encyclopedia of Genes and Genomes.

The asparagine-gene IDs were converted to NCBI IDs and run through KEGG pathway mapping software. Four genes with AS annotations (PANTHER) did not map to any KEGG pathway data, including the three identified as TSJT1 (*Glyma.07G257700*, *Glyma.13G279200*, and *Glyma.15G073100*) and the gene encoding a Wali7 domain-containing protein in *G. max* (*Glyma.15G105100*) (**Supplementary Table 3**). Three genes with AS annotations (*Glyma.11G171400*, *Glyma.11G170300*, and *Glyma.18G061100*) mapped to E.C.6.3.5.4, and two genes with ASPG annotations (*Glyma.17G238200* and *Glyma.04G042100*) mapped to E.C.3.5.1.1 on the alanine, aspartate, and glutamate (Ala-Asp-Glu) metabolism pathway in soybeans (gmx00250) (**Figure 6**; **Supplementary Table 3**). The same five genes were also mapped to the biosynthesis of the secondary metabolites pathway (gmx01110) and the full metabolic pathway known for *G. max* (gmx01100). The three known *G. max* AS genes that mapped *via* KEGG to gmx00250 also mapped to the biosynthesis of the amino acid pathway (gmx01230); the two ASPG genes that mapped to gmx00250 also mapped to the cyanoamino acid metabolism pathway (gmx00460) (**Supplementary Table 3**).

Using DNASTAR MegAlign Pro (v17.4.3), we aligned the following: the amino acid sequences for *Glyma.07G257700*, *Glyma.13G279200*, *Glyma.15G073100*, *Glyma.15G105100*, *Glyma.11G171400*, *Glyma.11G170300*, and *Glyma.18G061100*; AS full sequence in *G. max* (XP_003538618.1; 566 amino acids); and the AS *G. max* glutamine amidotransferase (GATase) domain type-2 (amino acids 2–185). **Supplementary Figure 2** shows the protein sequence alignment of the AS sequences DE in this study and the known AS amino acid sequence, including the functional domain GATase. Using this analysis, we identified that the TJST1 protein sequences lack the Cys at amino acid 2 in the protein sequence, which is the active site for GATase activity (**Supplementary Figure 2**). In the Wali7 domain-containing protein sequence encoded by *Glyma.15G105100*, there is Glu instead of the Cys at the GATase active site. Further, the binding site for l-glutamine (amino acid position 98) is an aspartate in the AS and the GATase domain sequences, but in the TJST1 and Wali7 domain-containing proteins, glutamate is encoded (**Supplementary Figure 2**).

#### 3.3.2 Alanine-related genes

Seven genes were found to be DE between East and West, six of which were downregulated (*Glyma.01G129400*, *Glyma.03G040600*, *Glyma.07G247900*, *Glyma.11G006500*, *Glyma.17G026200*, and *Glyma.18G021000*), and one (*Glyma.18G116900*) was upregulated in the West (**Figure 5B**; **Supplementary Table 2**). *Glyma.01G129400* was the most persistently downregulated gene, with 28 instances of downregulation out of the 30 DE datasets; this gene is predicted to be an alanine–glyoxylate aminotransferase 2 homolog 2 (mitochondrial) (E.C.2.6.1.44; **Figure 6**; **Supplementary Table 3**) in *G. max* and BLASTP identified alanine glyoxylate aminotransferase-like protein in *Medicago truncatula* as the most closely related protein (**Supplementary Table 2**). *Glyma.18G021000* was downregulated in the West in 26 of 30 datasets; this gene is uncharacterized in *G. max* but most closely related to an alanine–glyoxylate aminotransferase 2, (mitochondrial, fragment) in *Tupaia chinensis* (**Supplementary Table 2**).

*Glyma.18G116900* was the only gene upregulated in the West with alanine-related annotations that fit our stringent criteria (**Figure 5B**; **Supplementary Table 2**). This enzyme falls within the same pathway enzymatic element (E.C.2.6.1.44) but encodes a serine–glyoxylate aminotransferase-like protein in *G. max* (**Supplementary Table 3**). Two of the downregulated genes mapped to the Ala-Asp-Glu metabolism pathway *via* KEGG mapping: *Glyma.01G129400* and *Glyma.03G040600*. These two genes both encode alanine–glyoxylate aminotransferase 2 homolog 2 and homolog 3 and are also enzymatic components mapping to E.C.2.6.1.44 (**Figure 6**; **Supplementary Table 3**).

#### 3.3.3 Aspartate-related genes

In total, seven genes with “aspartate” in their annotations were found to be DE between East and West (p-value <0.01, |log_2_FC| ≥ 1.5, minimum 15 of 30 datasets) with a total of 163 instances of significant DE across all 30 datasets (**Supplementary Table 2**).

Four genes were found to be upregulated (*Glyma.04G042100*, *Glyma.05G007600*, *Glyma.17G238200*, and *Glyma.19G159300*); two of these genes encode ASPGs (E.C.3.5.1.1; **Figure 6**) and were present on the upregulated asparagine-related gene list (*Glyma.04G042100* and *Glyma.17G238200*) (**Supplementary Table 2**). *Glyma.05G007600* was upregulated in all 30 datasets; *Glyma.05G007600* encodes l-aspartate oxidase (E.C.1.4.3.16; **Figure 6**; **Supplementary Table 3**) in *G. max*. *Glyma.19G159300* was upregulated in 25 of 30 datasets; this gene encodes a lifeguard 4 protein in *G. max* and is most closely related to the gene encoding the glutamate-binding protein in *Arabidopsis thaliana* (**Supplementary Table 2**).

Three genes were largely downregulated in the West (*Glyma.02G015800*, *Glyma.14G111800*, and *Glyma.17G116500*; **Figure 5C**; **Supplementary Table 2**). *Glyma.14G111800* encodes an aspartate aminotransferase P2 (E.C.2.6.1.1) and was found to be downregulated in 15 of 30 DE datasets; however, 10 of these instances were in Saskatoon, and two and three instances were in Brandon and Morden, respectively. *Glyma.02G015800* was found to be downregulated in 16 of 30 datasets; this gene encodes fumatate hydratase 1 (E.C.4.2.1.2) in *G. max*. *Glyma.17G116500* was found to be downregulated across 28 of 30 datasets; this gene encodes broad specificity amino-acid racemase RacX in *G. max* and is homologous to aspartate-glutamate racemase family proteins in *Populus trichocarpa* and *A. thaliana* (**Supplementary Table 2**).

#### 3.3.4 Glutamate-related genes

Thirty genes with glutamate-inclusive annotations were found to be DE between eastern- and western-grown soybeans, which was made up of 13 upregulated genes and 17 downregulated genes (|log2FC| ≥ 1.5, p-value <0.01, in at least 15 of 30 DE datasets) (**Supplementary Table 2**). Of the upregulated glutamate-related genes, two were found to be upregulated across all 30 East *vs.* West DE datasets, both of which encode proline dehydrogenase *Glyma.13G049700* (proline dehydrogenase 2, mitochondrial) and *Glyma.19G043000* (proline dehydrogenase). *Glyma.19G111000* encodes a glutamate dehydrogenase 1-like protein and was the only gene among upregulated glutamate-related genes to map to an E.C. using KEGG:glutamate dehydrogenase (E.C.1.4.1.3) (**Figure 6**; **Supplementary Table 3**). *Glyma.13G233000*, which encodes glutamate receptor 2.7, was downregulated in the West across all 30 DE datasets (**Figure 5D**; **Supplementary Table 2**). A number of other genes were found to be downregulated across nearly all western-grown soybeans, including *Glyma.08G129600* (cationic amino acid transporter 1) (29), *Glyma.17G116500* (broad specificity amino-acid racemase RacX) (28), and *Glyma.03G069400* (δ-1-pyrroline-5-carboxylate synthase; ALDH18B3) (28) (**Figure 5D**; **Supplementary Table 2**). Four downregulated genes mapped to the Ala-Asp-Glu metabolism pathway (gmx00250): *Glyma.01G099800*, *Glyma.04G236900*, *Glyma.09G168900*, and *Glyma.18G041100*. *Glyma.01G099800*, another δ-1-pyrroline-5-carboxylate synthase (ALDH18B1), was downregulated in 20 of 30 datasets (**Figure 5D**; **Supplementary Table 2**); this gene mapped to E.C.1.2.1.88 class of oxidoreductases in the production of glutamate (**Figure 6**; **Supplementary Table 3**). *Glyma.04G236900* encodes a NADH-dependent glutamate synthase and was found to be downregulated in 15 datasets (**Figure 5D**; **Supplementary Table 2**) and mapped to the glutamate synthase enzymatic component (E.C.1.4.1.14) in the Ala-Asp-Glu pathway (**Figure 6**; **Supplementary Table 3**). *Glyma.09G168900* encodes a glutamate decarboxylase and was downregulated in 27 of 30 datasets (**Figure 5D**; **Supplementary Table 2**); KEGG mapping identified this gene as an enzyme component included in the Ala-Asp-Glu metabolism pathway, glutamate decarboxylase (E.C.4.1.1.15) (**Figure 6**; **Supplementary Table 3**). *Glyma.18G041100* is a known glutamine synthetase nodule isozyme and was found to be downregulated in 23 of 30 datasets; this gene mapped to the glutamine synthetase enzyme component (E.C.6.3.1.2) (**Figure 6**; **Supplementary Table 3**). For the full annotated list of DE glutamate-related genes and relative expression, see **Supplementary Table 2**.

#### 3.3.5 Oxaloacetate-related genes

A total of eight genes were identified to be DE between East and West, seven of which were upregulated (*Glyma.04G086300*, *Glyma.06G087800*, *Glyma.08G201200*, *Glyma.13G354900*, *Glyma.15G019300*, *Glyma.15G055600*, and *Glyma.18G261800*) and one of which was downregulated (*Glyma.01G008500*) (**Figure 5E**; **Supplementary Table 2**). From model-based data, NCBI identities of six of the oxaloacetate-related genes are NADP-dependent malic enzymes in *G. max*, five of which are upregulated (*Glyma.04G086300*, *Glyma.06G087800*, *Glyma.08G201200*, *Glyma.13G354900*, and *Glyma.15G019300*) and a single downregulated gene (*Glyma.01G008500*) (**Figure 5E**; **Supplementary Table 2**). One of the upregulated genes (*Glyma.15G055600*) is uncharacterized in *G. max*; however, BLASTP identified the most closely related protein to be a 2-oxoglutarate/malate translocator in *M. truncatula*. All six known malic enzyme genes (five upregulated and one downregulated) mapped to malate dehydrogenase (E.C.1.1.1.40), a component of the pyruvate metabolism pathway (gmx00620) and the carbon fixation in photosynthetic organism pathway (gmx00710) (map data not shown; see **Supplementary Table 3**). *Glyma.13G354900* (malic enzyme) was upregulated in the West across all 30 DE datasets, and four genes were found to be DE in nearly all 30 datasets: *Glyma.15G019300* was upregulated in 29 of 30 datasets, *Glyma.18G261800* was upregulated in 28 of 30 datasets, *Glyma.06G087800* was upregulated in 27 of 30 datasets, and *Glyma.15G055600* was upregulated in 26 of 30 datasets (**Figure 5E**; **Supplementary Table 2**).

#### 3.3.6 QTL analysis

**Figure 7:**
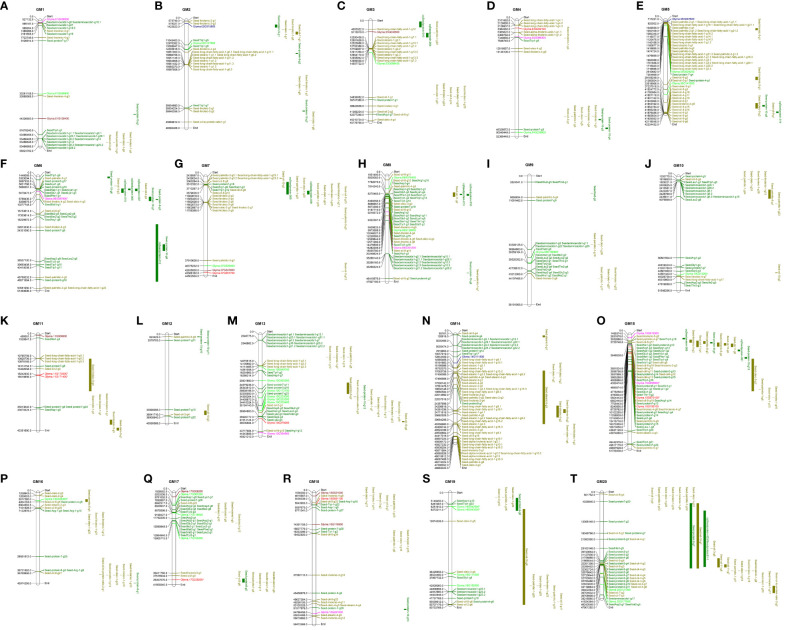
QTL maps for all 20 *Glycine max* chromosomes including all known protein and oil QTL information. Chromosome number is written above each map. Larger-spanning QTL regions are represented as bars to the right of each map; single-point QTLs (SNPs) are mapped directly onto the chromosome. Protein QTLs are in dark green; lipid QTLs are in mustard; asparagine-related genes are in red; alanine-related genes are in orange-red; oxaloacetate-related genes are in pink; aspartate-related genes are in royal blue; glutamate-related genes are in lime green; gene positions in cM are included in black text. Genes with an asterisk fall within spanning QTL regions. QTL, quantitative trait locus; SNPs, single-nucleotide polymorphisms. Chromosomes are in order from 1–20: **(A)** chromosome 1; **(B)** chromosome 2; **(C)** chromosome 3; **(D)** chromosome 4; **(E)** chromosome 5; **(F)** chromosome 6; **(G)** chromosome 7; **(H)** chromosome 8; **(I)** chromosome 9; **(J)** chromosome 10; **(K)** chromosome 11; **(L)** chromosome 12; **(M)** chromosome 13; **(N)** chromosome 14; **(O)** chromosome 15; **(P)** chromosome 16; **(Q)** chromosome 17; **(R)** chromosome 18; **(S)** chromosome 19; **(T)** chromosome 20.

QTL analysis was performed using the bottom-up gene lists to determine if any of the DE genes of interest fall within seed protein or oil QTLs. **Figures 7A–T** depicts *G. max* chromosomes 1–20 with known seed protein and oil QTLs mapped alongside the bottom-up genes of interest. Genes that are found within large-spanning QTL regions are denoted with an asterisk. **Supplementary Table 4** provides the map details for all QTLs and genes depicted in **Figure 7**; the green highlight in this table indicates regions that fall within a major spanning QTL. On chromosome 11, *Glyma.11G170300* (AS; 18,242,402 cM) and *Glyma.11G171400* (AS; 18,518,857 cM) fall within a large oil QTL, seed linoleic 5-g4 (10,969,418–25,595,388 cM) (**Figure 7K**). On chromosome 15, *Glyma.15G055600* (2-oxoglutarate/malate translocator-like protein; 4,350,474 cM) and the most persistently downregulated asparagine-related gene *Glyma.15G073100* (stem-specific protein TSJT1; 5,604,443 cM) fall within a large oil QTL, seed oil 11-g5 (4,148,354–5,633,343 cM) (**Figure 7O**; **Supplementary Table 4**). *Glyma.05G007600* (l-aspartate oxidase) is in close proximity to many oil QTLs on chromosome 5 (**Figure 7E**; **Supplementary Table 4**). Just outside of the QTL seed oil 11-g5 is *Glyma.15G105100* (stem-specific protein TSJT1; 5,604,443 cM), among the downregulated asparagine-related genes (**Figure 7O**; **Supplementary Table 4**). Within a large oil QTL on chromosome 16 called seed palmitic 6-g4 (3,002,525–4,148,354 cM) includes *Glyma.16G038300* (methionine synthase; 3,617,280 cM) (**Figure 7P**; **Supplementary Table 4**); this gene was found in the glutamate-related data to be upregulated in 26 of 30 East *vs.* West DE datasets (**Figure 5D**; **Supplementary Table 2**). Chromosome 17 has a large protein QTL, seed protein 9-g5 (38,930,849–40,629,216 cM), which includes one of the most persistently DE ASPG genes, *Glyma.17G238200* (ASPG; 39,357,077 cM). Two genes on chromosome 19, *Glyma.19G042900* (proline dehydrogenase 2; 6,251,912 cM) and *Glyma.19G043000* (proline dehydrogenase; 6,272,211 cM), fall within a very large seed protein QTL, seed protein 9-g6 (2,437,848–8,172,484 cM); these two genes were upregulated in 20 and 30 datasets, which of course makes *Glyma.19G042900* among the most persistently DE genes in the glutamate analysis.

## 4 Discussion

In this research, we investigated differences in the expression of genes between soybeans grown in East and West Canada in an effort to uncover DE genes and pathways that may contribute to the difference in seed protein content observed between the two locations over the past two decades (Canadian Grain Commission, 2022). Ten soybean genotypes were compared between East (Ottawa ON) and three different western locations (Morden MB, Brandon MB, and Saskatoon SK) in order to relieve genotypic and location biases for this large-scale RNA-seq and DE analysis. In this study, top-down (holistic) and bottom-up (keyword annotation search) approaches were used for the analysis of DE data to investigate the genes that are most consistently DE between East and West with putative roles influencing seed protein biosynthesis and accumulation.

### 4.1 East *vs.* West transcriptome variability

The PCA plots in **Figure 1** show a clear separation between variability of expression in the East from variability of expression across all three western locations, implicating that all lines across the western-grown soybeans are behaving similarly to the others from the West and all eastern-grown soybeans are behaving similarly to others in the East. These plots were constructed using RNA-seq variability as the principal components, and with these results, we observe a clear difference in expression variability between the two geographic areas (East and West). Considering the fact that RNA-seq variability was used to assess identical genotypes grown in four different environments, it can be confidently concluded that across all 10 lines, soybeans in the West show differential transcriptomics than eastern-grown counterparts. Each plot in **Figure 1** represents a different genotype; colored data points represent replicates from each location, and gray data points correspond with the data on all other plots (all other genotypes). It is evident that all samples from the West are behaving more or less the same across all 10 genotypes, and the same can be said for the East. Indeed, many factors, both genetic and environmental, cumulatively influence resultant seed protein content (Wang et al., 2019); thus, we used big-data ontology, pathway, and QTL analyses to refine genes that were consistently DE between East and West across 30 individual datasets. The clustering of the transcriptome data from the three West locations is clearly separate from the data from the East across all 10 genotypes, indicating that location biases are minimized.

### 4.2 Amino acid biosynthesis and seed protein, with a focus on the Ala-Asp-Glu pathway

Among enriched pathways, biosynthesis of secondary metabolites (gmx01110) was prominently DE between samples grown in the two regions. GO and KEGG analyses identified different amino acid biosynthetic pathways were differently regulated between East and West, including aromatic amino acids (GO:0009072; gmx00400), sulfur-containing amino acids (GO:0000096; gmx00270), glycine (GO:0006546), and glutamate aspartate and asparagine (GO:0006537; GO:0033345; GO:0008734; gmx00250). A focus on the Ala-Asp-Glu biosynthesis pathway (gmx00250) was chosen because of the relationship between nitrogen assimilates, asparagine, and seed protein at maturity. Previous investigations into the DE between vegetable soybean and grain soybean found that the Ala-Asp-Glu metabolic pathway was highly enriched, as well as fatty acid biosynthesis and metabolism, carbon (starch and sucrose) metabolism and transport, arginine and proline metabolism, and glycolysis/gluconeogenesis, all of which influence the attributes (including protein) of the resulting seed (Chen et al., 2022).

During embryo development in plants, sucrose provides a source of carbon, and glutamine and asparagine are the main nitrogenous assimilate sources (Rainbird et al., 1984). Asparagine plays a key role in the source (root nodules)–sink (seeds, mainly) translocation relationship (Lam et al., 1996). Asparagine has a relatively high nitrogen:carbon ratio and is biochemically stable, which make it ideal for nitrogen transport and storage. A careful balance exists between asparagine biosynthesis and degradation to maintain asparagine concentration. Asparagine represents up to 50% of the total free amino acids in the developing cotyledon (Hernández-Sebastià et al., 2005). AS is the major enzyme responsible for synthesizing asparagine. Typically, two or more AS genes are found in plants. High AS activity in the cotyledons of the germinating seed, as well as in mature root nodules, supports the idea that asparagine acts as a nitrogen transport system in legume plants (Lam et al., 1996). AS generates asparagine from aspartate by using glutamine or ammonia as a substrate for the transfer of the amide group to aspartic acid in an ATP-dependent reaction catalyzed by magnesium (Lea et al., 2007). AS proteins are categorized as either AS-A (a.k.a. AsnA) or AS-B (a.k.a. AsnB); AS-B family proteins, found in both prokaryotes and eukaryotes, can use both ammonia and glutamine as a nitrogen donor but prefer glutamine (Manhas et al., 2014). Glutamine-dependent AS is the main asparagine biosynthesis pathway in plants (Lam et al., 1996). ASPGs are ubiquitous across all domains of life. ASPG breaks down the isoaspartyl peptide bond in asparagine to aspartate and ammonia, which are then reassimilated through the glutamine synthase/glutamate synthase cycle (Gomes and Sodek, 1984; Haga and Sodek, 1987). In soybeans, ASPG activity is directly associated with a reduction in free asparagine by up to 18% while simultaneously increasing the amount of aspartate by up to 60% (Pandurangan et al., 2012). ASPG activity was also associated with a reduction in total nitrogen by 9%–13% and an increased concentration of seed oil by 5%–8% in soybeans (Pandurangan et al., 2012).

From this study, we observe differences in the expression of genes related to asparagine metabolism between soybeans grown in eastern and western Canada. The western-grown soybeans show downregulation of AS compared to eastern-grown soybeans (**Figure 5A**; **Supplementary Table 2**). This indicates that soybeans in the West are not synthesizing asparagine to the same degree as soybeans in the East, which may be directly attributed to the seed protein content at maturity. The western soybeans showed consistent upregulated expression of ASPG compared to the eastern counterparts. One of the most persistently upregulated ASPG genes, *Glyma.17G238200*, falls within a large protein QTL, seed protein 9-g5 (**Figure 7**; **Supplementary Table 4**). Further, the most persistently downregulated asparagine-related gene, *Glyma.15G073100* (stem-specific protein TSJT1), is within the major oil QTL, seed oil 11-g5 on chromosome 15 (**Figure 7**; **Supplementary Table 4**), another chromosome highly enriched for seed protein and oil QTLs. This might suggest that these genes are linked to seed protein and oil contents. AS and TSJT1 both have annotations that include “asparagine synthetase”; however, the sequence-based analysis uncovered a major difference in their protein sequences: TSJT1 protein sequences do not contain the GATase activity position 2 Cys, and the Wali7 domain-containing protein sequence has a Glu at amino acid position 2 (**Supplementary Figure 2**). AS genes *Glyma.11G171400*, *Glyma.11G170300*, and *Glyma.18G061100* are extremely downregulated in Brandon and Morden, but not found to be downregulated in Saskatoon (**Supplementary Table 2**). One TSJT1-encoding gene, *Glyma.15G073100*, was downregulated across all 30 datasets, while the other TSJT1-encoding genes were found to be downregulated mostly in Brandon and Morden (*Glyma.13G279200*) or Saskatoon (*Glyma.07G257700*).

The concentration of protein in mature soybeans is strongly associated with free asparagine in the plant during development, making it an ideal pathway for further investigation (Pandurangan et al., 2012). With the observations made in this study that ASPG is highly upregulated in the West and AS is highly downregulated in the West, it is entirely plausible that differences in asparagine metabolism are influencing the seed protein accumulation difference between eastern- and western-grown soybeans. It would be of interest to soybean breeding programs to consider increasing AS expression as an engineering target when designing high-protein soybean lines. AS1 overexpression in *A. thaliana* resulted in increased free asparagine levels and increased seed protein concentration (Lam et al., 2003). An increase in AS1 expression in soybean leaves had a positive correlation to seed protein concentration (Wan et al., 2006). In soybean roots, increased AS1 expression was correlated with an increased ratio of asparagine:aspartate in xylem sap headed to shoots, implicating more asparagine being transported to aerial tissues (Antunes et al., 2008).

Increased expression of asparagine aminohydrolases in western soybeans indicates that these plants are breaking down available asparagine to recycle the components, most specifically the nitrogen. When nitrogen is limited, hydrolyzing asparagine provides a source of nitrogen to be redirected into other processes. Because of the central intermediary relationship between alanine and/or serine and asparagine transamination (both amino acids can act as a substrate), increased ASPG expression, which leads to a reduction in freely available asparagine (Pandurangan et al., 2012), could logically be associated with equally proportionate increases in serine and alanine. Interestingly, in the data presented in this study, an increase in the gene encoding a serine–glyoxylate aminotransferase 2 (*Glyma.18G116900*) was observed, while multiple genes encoding alanine–glyoxylate aminotransferases (*Glyma.01G129400*, *Glyma.03G040600*, and *Glyma.18G021000*) were highly downregulated (**Figure 5B**; **Supplementary Table 2**). This means that different activities between two different enzymes of the same enzyme component (E.C.2.6.1.44) are simultaneously ongoing in western-grown soybeans, as presented in **Figure 6** by the half-blue-half-red box.

The asparagine synthesis pathway within the Ala-Asp-Glu metabolism pathway shows two other enzyme components that directly influence asparagine biosynthesis/metabolism: E.C.6.3.1.1 and E.C.3.5.1.38 (**Figure 6**). E.C.6.3.1.1 is an AS-A family AS that is an aspartate–ammonia ligase and was not found to be DE within our data. This is expected because plant AS is of the AS-B family of AS proteins and primarily uses glutamate as a N donor source rather than aspartate (Manhas et al., 2014). The KEGG pathway depicted in **Figure 6** KEGG shows enzyme components that are known to be in soybeans by highlighting respective boxes in green. E.C.3.5.1.38 appears to be prokaryotic in nature as indicated by available information on KEGG and BRENDA enzyme databases. It should be noted that as a result of the decrease in asparagine, western-grown soybeans could be compensating by increasing the expression of other nitrogen-rich amino acid (arginine and lysine) metabolizing enzymes (Pandurangan et al., 2012), which were not covered in our bottom-up analysis and might serve as an interesting area for further research.

Alanine is one of the central intermediates in amino acid metabolism and a substrate of asparagine transaminase, the enzyme responsible for transferring the α-amino group between asparagine and glycine, alanine, serine, and homoserine (Pandurangan et al., 2012; Zhang et al., 2013; Gaufichon et al., 2015). There is a central intermediary relationship between asparagine transamination and alanine and/or serine in that both amino acids can act as a substrate (Pandurangan et al., 2012). Overall, the results from the alanine investigation indicate the downregulation of alanine-related genes, with six downregulated genes and one upregulated gene common across at least 50% of the DE datasets (**Figure 5B**; **Supplementary Table 2**). Significant downregulation of *Glyma.01G129400*, *Glyma.03G040600*, and *Glyma.18G021000*, three alanine–glyoxylate aminotransferases, was observed in the West (**Figure 5B**; **Supplementary Table 2**). An increase in ASPG leads to a decrease in asparagine and potentially an increase in alanine, which may in part explain the downregulation of alanine-related genes as a whole (**Figure 5B**). In **Figure 6**, E.C.2.6.1.44 is both up- and downregulated in the West (half-blue-half-red box). Western-grown soybeans appear to be upregulating the alanine-related gene, *Glyma.18G116900*, encoding serine–glyoxylate aminotransferase 2 (E.C.2.6.1.44), which is also involved in serine-pyruvate transaminase activity (GO:0004760). Simultaneously, these soybeans are downregulating the expression of two alanine–glyoxylate aminotransferases (*Glyma.01G129400* and *Glyma.03G040600*) (**Figure 5B**; **Supplementary Table 2**). In addition to the Ala-Asp-Glu pathway, enrichment for the cyanoamino acid metabolism pathway (gmx00460) is a result of the two ASPG genes (*Glyma.17G238200* and *Glyma.04G042100*) (**Supplementary Table 3**). High-protein soybean genotypes were found to have higher amounts of free asparagine and alanine in developing embryos than in low-protein genotypes (Hernández-Sebastià et al., 2005). Further, freely available 3-cyanoalanine was found to be highly correlated with seed protein and/or oil in soybeans (Wang et al., 2019). In an investigation into the genetic shift in soybeans over 24 years, it was found that newer soybean cultivars had a decrease in seed protein, alanine, and serine (de Borja Reis et al., 2020). The relationship between alanine, serine, and asparagine in soybean seed protein accumulation remains elusive, and further investigations into the relationship between these amino acids and protein should be explored.

The aspartate-family amino acid sub-pathway functions as a regulatory metabolic link with the tricarboxylic acid (TCA) cycle, biologically significant under extreme stress conditions, which deplete cellular energy (Galili, 2011), making it an essential metabolite for plant growth and stress acclimation (Han et al., 2021). Aspartate-family amino acids (lysine, threonine, methionine, and isoleucine) are synthesized in plants using aspartate as a central amino acid (Galili, 2011). In this study, *Glyma.05G007600* encoding l-aspartate oxidase (E.C.1.4.3.16) was significantly upregulated in the West. This gene is physically close on chromosome 5 to many known oil QTLs (**Figure 7**; **Supplementary Table 4**). Chromosome 5 is highly enriched for QTL influencing protein and oil; however, the molecular mechanisms driven by these loci remain largely unknown (Wang et al., 2019). The closeness in proximity suggests that they are tightly linked, and following recombination, it would be advantageous for these genes to remain together in future progeny.

*Glyma.19G159300* (lifeguard 4 protein in *G. max*) was upregulated in 25 of 30 datasets (**Figure 5D**); this gene is most closely related to the gene encoding the glutamate-binding protein in *A. thaliana* and is also closely related to the inhibitor of the apoptosis-promoting BAX1 protein in *A. thaliana* (**Supplementary Table 2**). Further investigations into the glutamate binding potential of this gene and the putative role it plays in signaling would be of merit to understanding the reason(s) for significant upregulation of *Glyma.19G159300*. It is likely that this protein plays a role in signaling and proliferation, potentially inhibiting apoptosis. Increasing expression of genes related to glutamate signaling and apoptosis suggests that western-grown soybeans are differently controlling cell proliferation compared to those in the East.

Glutamate has a remarkably wide range of biological roles because of the central position it plays in metabolism. It is suggested that glutamate compensates for the reduction in freely available asparagine by serving as a metabolite (nitrogen) storage molecule and behaves as an organic nitrogen signal in seedlings (Gutiérrez et al., 2008; Pandurangan et al., 2012). This study uncovered 30 glutamate-related genes that are DE in at least 50% of the datasets. Within the glutamate data, genes for proline dehydrogenases (*Glyma.13G049700*, *Glyma.19G042900*, and *Glyma.19G043000*) were among the most upregulated in the West. Proline dehydrogenase catalyzes the oxidation of l-proline to δ1-pyrroline-5-carboxylate, which provides a source of free electrons for transport (Servet et al., 2012). The two proline dehydrogenase genes on chromosome 19 both fall within a major seed protein QTL, seed protein 9-g6 (**Figure 7**). Proline dehydrogenase catabolizes proline while simultaneously playing roles in energy, shuttling redox potential, and production of reactive oxygen species (ROS) to reach cellular homeostasis, adapt to the environment, and carry out physiological and pathological processes (Servet et al., 2012). A methionine synthase (*Glyma.16G038300*) was also highly upregulated in the West, which ultimately influences the sulfur-containing amino acid content of the developing seed. Methionine synthase is responsible for catalysis of 5-methyltetrahydropteroyltri-l-glutamate and l-homocysteine into l-methionine + tetrahydropteroyltri-l-glutamate in the synthesis of methionine and glutamate (Whitfield et al., 1970), a unique feature of some organisms that are able to convert Cys to Met under specific circumstances (Brosnan and Brosnan, 2006). Upregulation of this methionine synthase gene may suggest that western-grown soybeans are increasing glutamate in an attempt to compensate for a lack of free asparagine. If, coincidently, this also results in more Met production from l-homocysteine, the overall protein quality (in terms of 11S and 7S globulins) could be improved. Improved seed protein quality is an important consideration for soybean agriculture, particularly in regions where environmentally influenced decreases in seed protein levels are prominent (i.e., western Canada), and western-grown soybeans were found to have higher 11S:7S values than eastern-grown soybeans (Cober et al., 2023).

Also within the glutamate data are a number of DE glutamate receptor genes both upregulated (*Glyma.07G226400*, *Glyma.10G213200*, *Glyma.13G049700*, *Glyma.13G093300*, *Glyma.13G172100*, and *Glyma.13G233400*) and downregulated (*Glyma.13G233000*, *Glyma.13G233300*, and *Glyma.17G067200*) in the West (**Figure 5D**; **Supplementary Table 2**). This points to specific genes involved in DE glutamate signaling between eastern- and western-grown soybeans. The specific ligands for these receptors would be an interesting area for further research on these genes.

As previously stated, the Ala-Asp-Glu metabolism pathway coordinates a metabolic link to the TCA cycle, directly influencing energy production or depletion (Galili, 2011). Oxaloacetate is both a product and a beginning component of the TCA cycle; levels of oxaloacetate give an indication of the ongoing level of energy metabolism. The data uncovered in this study indicate major upregulation of oxaloacetate-related genes in western-grown soybeans; of eight significantly DE genes, seven were upregulated, and one was downregulated (**Figure 5E**; **Supplementary Table 2**). All of the DE genes related to oxaloacetate are malic enzymes (E.C.1.1.1.40, pyruvate metabolism pathway gmx00620; **Supplementary Table 3**), with the exception of *Glyma.15G055600*. *Glyma.15G055600*, a 2-oxoglutarate/malate translocator-like protein, was found within the oxaloacetate-related gene list (**Supplementary Table 3**) and the only oxaloacetate-related gene that does not map to any pathway using KEGG (**Supplementary Table 4**). However, *Glyma.15G055600* falls within the same major oil QTL as one of the most persistently downregulated asparagine-related genes (*Glyma.15G073100*), seed oil 11-g5 on chromosome 15 (**Figure 7**; **Supplementary Table 4**).

Malic enzyme is one of the key enzymes linked to fatty acyl chain biosynthesis. l-Malic acid was found to be highly correlated with protein and oil contents in soybeans (Wang et al., 2019). A significant amount of pyruvate, the precursor of acetyl-CoA synthesis for lipid biosynthesis, is produced as a result of malic enzyme activity in soybeans (Allen and Young, 2013). In western-grown soybeans in this study, malic enzyme activity was highly upregulated, which suggests that these plants are likely producing higher levels of acetyl-CoA for fatty acid production. Because of this, malic enzyme activity is almost certainly one of the regulatory mechanisms underlying the inverse relationship between seed protein and oil contents in soybeans. This mechanism serves are an optimal target for genetic engineering/control of the decision between lipid and protein biosynthesis. Increasing expression of malic enzyme would make a molecular conduit to directing nitrogen and carbon toward lipid biosynthesis and away from protein biosynthesis, serving as a molecular switch for the accumulation of major seed storage biomolecules (Morley et al., 2023).

### 4.3 Sulfur-containing amino acid biosynthesis and seed protein

The ontologies sulfur amino acid metabolic process (GO:0000096), sulfur compound biosynthetic process (GO:0044272), sulfur compound metabolic process (GO:0006790), iron-sulfur cluster binding (GO:0051536), and more were all enriched and overrepresented within the top-down GO analysis (**Figure 3**; **Supplementary Table 1**). Further, the sulfur-containing amino acid Cys and Met metabolism pathway (gmx00270) was enriched within the DE data. These observations that transcription of genes related to the biosynthesis of sulfur-containing amino acids (Cys and Met) are DE between East and West likely play a role in the differences in seed protein quality seen between western- and eastern-grown soybeans (Cober et al., 2023). Sulfur-containing amino acids are essential to the formation of 11S storage proteins (glycinins) in soybean. Sedimentation coefficients (0.5-M ionic strength) are used to categorize seed storage proteins into 2S, 7S, 11S, and 15S fractions, of which the 11S and 7S fractions account for the majority of seed storage proteins (40% and 30% of seed storage protein, respectively) (Peng et al., 1984; Tsukada et al., 1986). Cys and Met are limited resources in soybeans, and tight control of biosynthesis of these amino acids is advantageous to glycinin production in soybeans. Because Cys and Met are vital to glycinin biosynthesis, the genes influencing expression and accumulation of sulfur-containing amino acids very likely influence glycinin accumulation (nutrient reservoir activity) and therefore seed protein content.

### 4.4 Other influential pathways on seed protein

Enriched among the data were many other pathways of interest That almost certainly influence seed protein content, including fatty acid metabolic processes (fatty acid biosynthetic process GO:0006633; lipid transport GO:0006869; lipid binding GO:0008289; gmx01212), circadian rhythm (circadian rhythm GO:0007623; regulation of circadian rhythm GO:0042752; gmx04712), nutrient storage (nutrient reservoir activity GO:0045735), and carbohydrate metabolism (carbohydrate metabolic process GO:0005975; carbohydrate-binding GO:0030246; regulation of carbohydrate metabolic process GO:0006109) (**Figures 3**, **4**; **Supplementary Table 1**). Western locations in this study are also further North than the Eastern locations and as a result experience longer photoperiods; the circadian rhythm of soybeans in these locations is almost certainly going to exhibit differences. An insertion/deletion in *Glyma.20G85100*, a circadian clock gene, was found to nearly perfectly correspond with high/low protein alleles for a QTL on chromosome 20, cqSeed protein-003 (Fliege et al., 2022). While *Glyma.20G85100* was not DE in our data, genes with circadian rhythm among their ontologies were overrepresented in both upregulated (20) and downregulated genes (23) (**Figure 3**; **Supplementary Table 1**). Response to UV (GO:0009411) is upregulated in western-grown soybeans, likely in response to reduced cloud cover in the prairies. Ontologies for microtubule cytoskeleton organization (GO:0000226), microtubule motor activity (GO:0003777), cytokinesis by cell plate formation (GO:0000911), cell wall biogenesis (GO:0042546), cell cycle (GO:0007049), and spindle assembly (GO:0051225) are highly enriched in the upregulated genes in our study (**Figure 3**, **Supplementary Table 1**). Soybeans in western Canada were found to be significantly taller than eastern-grown soybeans (Cober et al., 2023), which is likely influenced by these genes, suggesting the plants are spending more energy increasing in height than producing/filling seeds. Genes related to water stress were also found to be DE between East and West (**Figure 3**); response to desiccation (GO:0009269), water transport (GO:0006833), response to water depravation (GO:0009415), and water channel activity (GO:0015250) are all likely the result of the lower precipitation and lower relative humidity in the West.

Protein and oil compete for space in the seed, which results in a pushing/pulling relationship between these seed storage macronutrients (Breene et al., 1988; Clemente and Cahoon, 2009). Previous investigations into seed protein, oil, and yield identified an influential QTL on chromosome 20 between Satt496 and Satt239 (Chung et al., 2003); however, there were no DE genes found between these markers in our data. The relationship between seed storage and metabolism is entangled by the upstream carbohydrate metabolism decision-making steps that lead to protein and/or oil biosynthesis (40% and 20%, respectively) while also maintaining a proportion (~35%) of the seed space for stored carbohydrates (Liu, 1997). Seed storage proteins (glycinins, vicilins, and cupins) have the ontology nutrient reservoir activity (GO:0045735). Nutrient reservoir activity (GO:0045735) was overrepresented in the top-down downregulated genes but underrepresented (though present) in the upregulated GO enrichment (**Supplementary Table 1**). Known genes for glycinins and β-conglycinins (*Glyma.03G163500* GY1, *Glyma.03G163500* GY2, *Glyma.19G164900* GY3, *Glyma.10G037100* GY4, *Glyma.13G123500* GY5, *Glyma.10G246300* CG-1 α′1, *Glyma.20G148400* CG-2 α2, *Glyma.20G148300* CG-3 α1, *Glyma.20G146200* CG-4 β1, and *Glyma.20G148200* CG-4 β2) were not found to be significantly DE across a majority of lines but were found to be DE in some lines (**Supplementary Table 1**). β-Conglycinin genes are reported to only be expressed in seeds in early embryogenesis; transcription spikes at mid-maturation and decreases before dormancy (Harada et al., 1989). These genes are not expressed in cotyledons or at maturity; thus, it is reasonable that we do not see notable DE between East and West in the present data; leaf tissue at the R5 (seed filling) stage was used for RNA-seq. A similar investigation into RNA-seq analysis of soybean pod data would provide further information into the DE of glycinins and β-conglycinins. The findings in this study share similarities with a similar study conducted in China on high and low seed protein varieties, which found DE of genes involved in the biosynthesis of amino acids and secondary metabolites, carbon metabolism, lipid metabolism, phenylpropanoid biosynthesis, and plant hormone signal transduction (Xu et al., 2022), although we examined DE of individual varieties across geographies and not DE between varieties.

## 5 Conclusions

In this work, we identified genes persistently DE in 10 soybean varieties grown in three different locations in western Canada compared to an eastern Canada location. We pinpoint genes within specific metabolic processes that are likely key players in reduced protein content observed in western-grown soybeans, most pertinently genes encoding AS and ASPG. By investigating the differences in the expression of genes underlying nitrogen assimilation during seed development in soybeans grown in East and West Canada, we offer valuable information on the impact of geographic location on this pathway as well as potential avenues for breeding improvement opportunities. Further investigations into the lipid biosynthetic pathway, sulfur-containing amino acid biosynthesis pathway, and aromatic amino acid biosynthesis pathway would all likely provide key information into the differences in metabolic orchestration influenced by environment.

## Data availability statement

The original contributions presented in the study are included in the article/**Supplementary Material**. Further inquiries can be directed to the corresponding author.

## Author contributions

JH: Data curation, Formal Analysis, Methodology, Validation, Visualization, Writing – original draft, Writing – review & editing. MS: Conceptualization, Formal Analysis, Methodology, Writing – review & editing. GZ: Data curation, Formal Analysis, Software, Visualization, Writing – review & editing. MC: Data curation, Writing – review & editing. DL: Data curation, Methodology, Writing – review & editing. RM: Data curation, Writing – review & editing. KD: Data curation, Writing – review & editing. TW: Data curation, Resources, Supervision, Writing – review & editing. MH: Data curation, Writing – review & editing. BB: Data curation, Writing – review & editing. AH: Data curation, Resources, Writing – review & editing. FL: Data curation, Software, Writing – review & editing. AG: Formal Analysis, Supervision, Writing – review & editing. EC: Conceptualization, Formal Analysis, Funding acquisition, Methodology, Project administration, Resources, Writing – review & editing. BS: Conceptualization, Data curation, Formal Analysis, Funding acquisition, Investigation, Methodology, Project administration, Resources, Software, Supervision, Validation, Visualization, Writing – review & editing.

## Supplementary material

Supplementary Table 1 Top-down analysis of up- and downregulated genes in each line-location analysis, and cumulatively DE genes. **Supplementary Table 2**: Bottom-up analysis summarizing asparagine, alanine, aspartate, glutamate, and oxaloacetate DE data. **Supplementary Table 3**: KEGG pathway mapping information for top-down and bottom-up analyses. **Supplementary Table 4**: MapChart chromosome map data for *G. max* chromosomes 1–20. **Supplementary Figure 1**: High resolution image of top-down analysis KEGG pathway map (gmx01100). **Supplementary Figure 2**: Amino acid sequence alignment of AS-related proteins.
