# Supplementary figures and images for: Differential gene expression provides leads to environmentally regulated soybean seed protein content
Source: Front Plant Sci. 2023 Sep 18;14:1260393. doi: 10.3389/fpls.2023.1260393 (PMC10544915; doi:10.3389/fpls.2023.1260393)

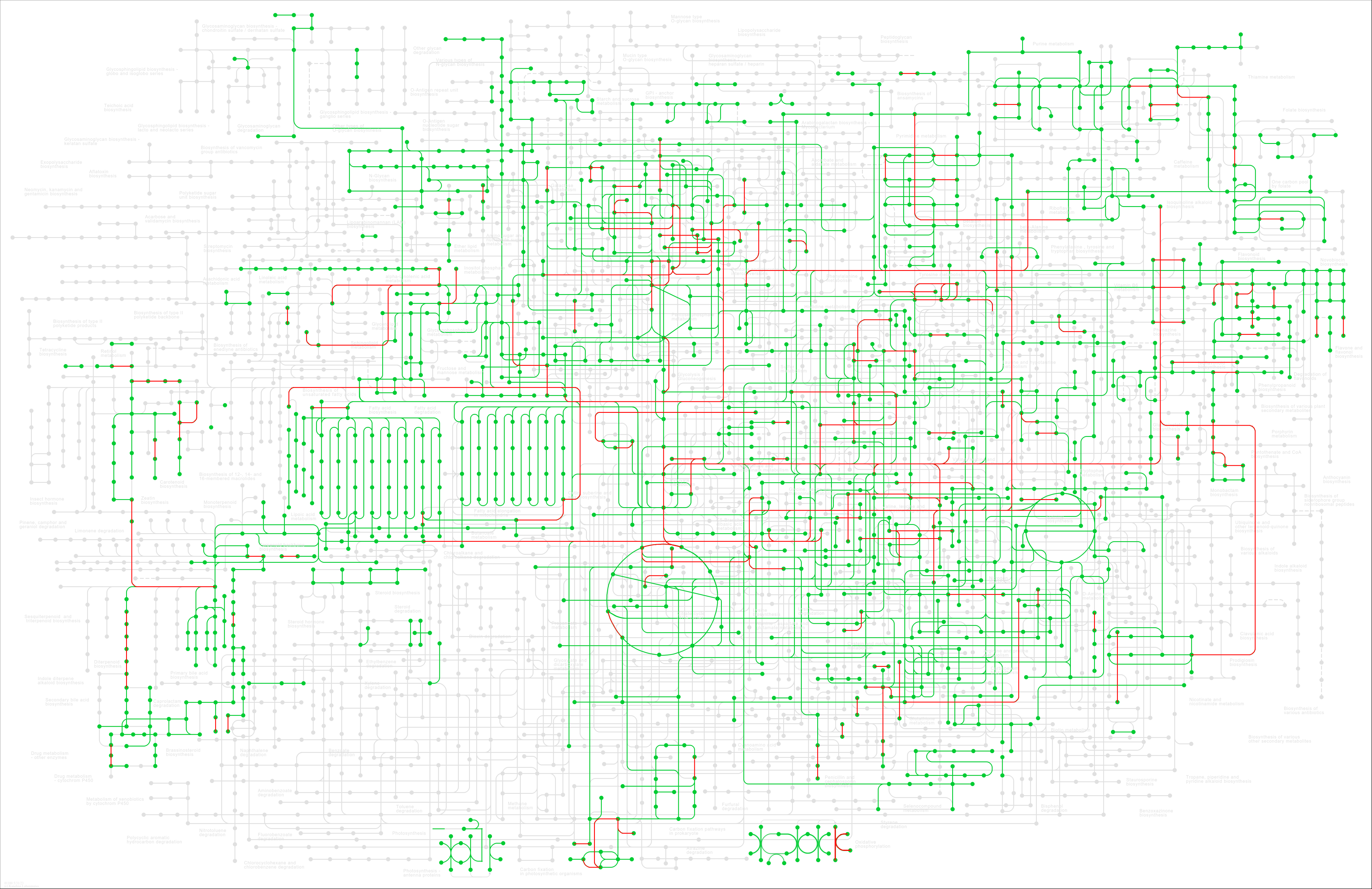

Supplement: Supplementary file 5 [file Image_1.jpeg]
